# Psychometric Properties of the Eating Disorder Inventory-3 (EDI-3) in Chilean Youth

**DOI:** 10.3389/fpsyg.2022.806563

**Published:** 2022-03-01

**Authors:** Paula Lizana-Calderón, Claudia Cruzat-Mandich, Fernanda Díaz-Castrillón, Jesús M. Alvarado, Emilio J. Compte

**Affiliations:** ^1^Centro de Estudios de la Conducta Alimentaria (CECA), Escuela de Psicología, Universidad Adolfo Ibáñez, Santiago, Chile; ^2^Facultad de Psicología, Universidad Complutense de Madrid, Madrid, Spain

**Keywords:** Eating Disorder Inventory-3 (EDI-3), bifactor exploratory structural equation modeling (ESEM), eating disorders (ED), psychometric procedures, eating disorders–diagnosis, therapy

## Abstract

The aim of this study was to analyze the psychometric properties of the Eating Disorder Inventory (EDI)-3 test to evaluate eating disorders in young Chilean population. Methods: The sample consisted of 1,091 Chilean adolescents and young people (i.e., 476 men and 615 women) between 15 and 28 years old, from the metropolitan region, and four regions from the coast and south-central zone of the country. The reliability and factorial structure of the instrument were analyzed, replicating the confirmatory factor analyses of Brookings et al. (2020), evaluating four additional models that included bifactor exploratory structural equation modeling (ESEM), bifactor, and two-bifactor. Results: A majority of the subscales presented alphas and omegas equal to or greater than 0.70, with the exception of asceticism (α = 0.543, ω = 0.552) and interpersonal alienation (α = 0.684, ω = 0.695) scales, which are consistent with the values of the Spanish and Mexican non-clinical samples. The best fit indices were obtained by the ESEM two-bifactor model, with twelve specific factors corresponding to the EDI-3 subscales and two general orthogonal factors (i.e., risk subscales and psychological subscales), consistently with the theoretical basis.

## Introduction

Eating disorders (EDs) are serious psychological problems, with high mortality and poor prognosis, strongly associated with thinness typical ideal of contemporary western societies (American Psychiatric Association, 2014). A recent systematic review (Galmiche et al., 2019), which includes 94 studies published in English or French, between 2000 and 2018, shows that the mean lifetime prevalence of EDs and their ranges in women was 8.4% [3.3–18.6%] and in men 2.2% [0.8–6.5%]. The mean prevalence in the last year and its range corresponded to 2.2% [0.8–13.1%] for women and 0.7% [0.3–0.9%] for men. The weighted means of the point prevalence and their ranges were 5.7% [0.9–13.5%] for women and 2.2% [0.2–7.3%] for men. When analyzing by continent, it stands out that America has the highest point prevalence mean, with 4.6% [2.0–13.5%]. The authors also incorporated 27 other studies that revealed that the mean point prevalence of any ED is 19.4% [6.5–36.0%] in women and 13.8% [3.6–27.1%] in men. The increase in point prevalence in the period studied was 3.5% between 2000 and 2006 and 7.8% between 2013 and 2018. An important study carried out with a sample of 36,309 people in the United States (Udo and Grilo, 2018) indicates that the ED would be linked to a significant deterioration in psychosocial functioning and that there were relevant associations between binge ED and extreme obesity, which, together with the high prevalence, would constitute a major public health problem.

The age of onset of EDs is earlier in anorexia nervosa (AN), on average before the age of 22 years. In bulimia nervosa (BN), it would appear before the age of 24 years. Due to the changes in the DSM-5 diagnostic criteria, there has been an increase in AN and BN and a decrease in diagnoses not specified (EDNOS) or other diagnoses (OSFED) (Lindvall Dahlgren et al., 2017).

In Chile, Correa et al. (2006) described that EDs have become chronic and, in recent years, have spread to men and different socioeconomic levels. Urzúa et al. (2011) observed significant differences between adolescents who attended public schools, showing a greater drive for thinness DT than those who attended private schools. In turn, a high-risk factor is the initiation of unsupervised diets at an early age, especially in overweight or obese young people, who have shown greater DT and body dissatisfaction (BD) (Contreras et al., 2015).

It was identified that the age of greatest risk of developing ED would be 16 years in women and 17 years in men (Zapata et al., 2018). Men may have a later onset of ED because pubertal changes are later than those observed in women (Salas et al., 2011).

For all the above, it is important to have standardized instruments that allow an early detection of EDs and is necessary to take a test that provides information on the risks in normal populations, especially adolescents, and also, in young adult populations, in whom high rates of maintenance of EDs are observed (Treasure et al., 2011). In addition, the instruments must be sensitive in the populations where they are used especially because EDs are strongly related to a social factor, such as DT, and the internalization of this ideal of beauty, especially in female adolescents (Mellor et al., 2008; Caqueo-Urízar et al., 2011; Cash and Smolak, 2011), and because Chilean families seem to exert a greater influence than another families on BD compared with that in peers influence (Mellor et al., 2008).

Although there are numerous instruments that allow the evaluation and diagnosis of EDs (Losada and Marmo, 2013), it is probably the Eating Disorders Inventory (EDI) that has been the most widely used to standardize the self-reports that measure the psychological symptoms associated with AN, BN, and other EDs (Nevonen et al., 2003; Clausen et al., 2009). The original EDI (Garner et al., 1983) that was revised in Garner (1991) covered a total of 64 items that were organized into eight subscales. The next version, EDI-2, was made up of 11 subscales derived from 91 items, against which it is possible to choose six response alternatives ranging from always to never agree.

From EDI-2, a new version called EDI-3 was created (Garner, 2004). New statistical analyses were carried out, generating new groupings of the items in the scales (item 71 was left out of the computations), and a new scoring system was incorporated which includes greater variability of the evaluated elements (i.e., 6-point Likert scale, which is scored between 0 and 4 points, and it was 0–3 points earlier). Thus, the EDI-3 scales seek to measure more reduced and discriminative constructs than the previous version.

The EDI-3 consists of 12 main scales and 6 indices. Three of the main scales are called risk scales, namely, DT, bulimia (B), and BD. The remaining nine scales, namely, low self-esteem (LSE), personal alienation (PA), interpersonal insecurity (II), interpersonal alienation (IA), interoceptive deficits (ID), emotional dysregulation (ED), perfectionism (P), asceticism (A), and maturity fears (MF) assess psychological aspects especially associated with the development and maintenance of ED. The EDI-3 also allows grouping some scales into six indices called risk of ED (DT + B + BD) and ineffectiveness index (LSE + PA), which account for a low personal assessment and a feeling of emotional emptiness related to a deficit in the constitution of identity. A third index is interpersonal problems (II + IA). It evaluates the possibility of the individual to trust interpersonal relationships, and the belief that these are tense and disappointing, so it has a predictive value for poor response to treatment. Another index is affective problems (ID + ED). It refers to difficulties in discriminating emotional problems and in expressing emotions appropriately. This element appears as a relevant factor in the maintenance of ED and therefore is one of the main goals of the therapy. Another index refers to excess control (P + A), which measures the desire for perfection through self-sacrifice, pillars of the ED generally resistant to change.

Another index is the general psychological maladjustment (i.e., sum all the psychological scales), which would allow predicting the results of the treatment, measuring the pattern of responses of the subject and indicating high levels of psychopathology. In addition, the EDI-3 has three scales, namely, validity, inconsistency, and infrequency (it refers to the responses that maximize the pathology, which is infrequent in the subjects of the clinical sample), and negative impression (it refers to the responses in which the subject chooses the most extreme options with the greatest symptoms), allowing the analysis of response patterns that suggest a bias in the results.

The EDI-3 has been validated with large samples in multiple languages and countries (Garner et al., 2010; Clausen et al., 2011; Nyman-Carlsson et al., 2015; Dadgostar et al., 2017), which has made it possible to develop scales for use in clinical and non-clinical populations and not only at risk, as was the EDI-2.

Only the EDI-2 psychometric properties have been analyzed in the Chilean population (Urzúa et al., 2009).

The EDI-3 Spanish version validations (Garner et al., 2010) have been carried out in various countries. In Mexico, it was carried out with a clinical sample in more than 500 women with a diagnosis of EDs (Unikel et al., 2006), who, through the principal component analysis (i.e., varimax rotation), conclude that the factorial structure corresponds to six factors, including 36 items, which explain 56% of the total variance. In Peru, the Spanish version was adapted using a sample of more than 600 people (Infantes, 2015). Another validation was carried out in Argentina with a sample of more than 700 female adolescents from the general population, who, based on an exploratory factor analysis of the risk and psychological scales separately, conclude that only the structure of the risk scales would be equivalent to the original version (Rutsztein et al., 2013). However, none of these validations checks the fit of the items to their respective factors (i.e., subscales), by means of the confirmatory factor analysis. Clausen et al. (2011) carried out the first evaluation of the first- and second-order factorial structure of the EDI-3 in its Danish language version, with a sample of 561 adult patients and a control group of 878 adult women, obtaining a good fit to a model of two second-order factors, one of risk and the other made up of psychological disorders, which supports the original structure proposed by Garner (2004). From these analyses, Brookings et al. (2020) replicated the evaluation of the second-order two-factor model, this time for the English version, in a clinical sample of 1,206 female patients aged between 11.4 and 74.3 years (mean 22.6 and SD 8.9 years) and test alternative models. The authors divided the sample into two subsamples. With the first, they evaluated the fit of the models proposed *a priori* and models proposed *a posteriori*. With the models tested in sample 1, a cross-validation was performed using sample 2. Finally, the models with the best fit were analyzed using the full sample. The model that presented the best fit was a model of 12 correlated factors and a second general factor orthogonal to the content factors, which covers the 90 items, considering five pairs of correlated errors, which correspond to items that refer to the same content, and either directly or inverse.

Based on the antecedents raised about the EDI-3 factorial structure, non-existent for the Spanish version, the aim of this study was to analyze the psychometric properties of the Eating Behavior Inventory (EDI-3) in a non-clinical population of young Chileans, replicating the analyses carried out by Brookings et al. (2020) and evaluating alternatives using Exploratory Structural Equation Modeling (ESEM).

## Materials and Methods

### Participants

The initial sample consisted of 1,346 students. 255 cases were discarded because they presented systematic missing values in the sociodemographic variables and in the items of the instrument. The eliminated cases were compared with the definitive cases and did not present a difference in the proportion of men and women (χ^2^ = 0.007; gl = 1; *p* = 0.933), nor in the mean body mass index (BMI, *t* = -1.411; gl = 1,301; *p* = 0.158). Only a difference in the mean age of 0.492 years (5.9 months) was observed in favor of the final sample (*t* = 2.782; gl = 1,344; *p* = 0.005), which would not be substantively relevant. Thus, the sample was made up of 1,091 Chilean adolescents and young people, of which 476 were men (43.6%) and 615 women (56.4%). The ages fluctuated between 15 and 28 years, with an average of 19.1 years (*SD* = 2.52) and 46.1% were between 15 and 18 years and the rest 19 years or more. The young people came from the Metropolitan Region and four regions from the coast and south-central zone of the country. Participants were selected by non-probability sampling by quotas. The minimum size of the sample was determined according to Soper (2015), considering α = 0.05; 1−β = 0.8; 14 latent variables, 90 observed variables, and anticipated effect size of 0.14, yielding a minimum sample size for the model structure of 274 and a minimum size for detecting the effect size of 1,110 subjects.

The BMI (weight kg/height m^2^) showed an average of 22.5 (*SD* = 3.1). According to this parameter, 2% were obese, 6.4% underweight, 16% overweight, and 82.2% had a normal nutritional state.

Regarding family history, 52.9% had a history of overweight, 45.4% of diabetes mellitus, and 41.6% of hypertension.

The students participated voluntarily in this study, without receiving compensation in return. A total of 338 participants (31%) were in secondary education, while 735 (67.4%) were in university studies (18 cases with missing values: 1.65%), including 25 different undergraduate programs, addressing all knowledge areas.

### Procedures

The participants were contacted through secondary and university educational institutions. In each of them, an institutional authorization was obtained, and the students were then evaluated in their educational entities. Consent was requested from adults, and in case of minors, consent was requested from parents and assent from students, explaining the aims of the study, the type of collaboration that was requested, and the guarantees of confidentiality, anonymity, and voluntariness This consent was approved by the Bioethics Committee of the National Commission for Scientific and Technological Research of Chile, CONICYT, and the students kept a copy of it. All questionnaires were anonymous.

The students answered the Eating Disorder Inventory 3 (EDI-3) and, in addition, a sociodemographic questionnaire that was used to characterize the sample and that included variables such as sex, age, weight, height, occupation and educational level, and health history of the parents and relatives of the participant.

### Data Analysis

For the evaluation of the psychometric properties of the EDI-3, the internal consistency of its scores for each subscale was first analyzed using the reliability coefficient Cronbach’s alpha and McDonald’s omega because it is not possible to assume that the items are tau-equivalent (Trizano-Hermosilla and Alvarado, 2016).

The adjustment was evaluated using the confirmatory factor analysis (CFA, Weighted Least Square Mean and Variance Adjusted Estimators, WLSMV, for ordinal data) using the models proposed by Brookings et al. (2020): Model 1: 12 correlated factors; Model 1A: 12 correlated factors and select correlated errors, corresponding to five pairs of items: 2 and 12 from BDI; 13 and 43 of P; 19 and 20 of BDI and PA, respectively; 69 and 73 of II; and 72 and 83 of ED (J. Brookings, personal communication, January 11, 2021); Model 2: 12 correlated factors with 10 correlated errors for inconsistency scale items; Model 3: two second-order factors (risk scales and psychological scales); Model 3A: two second-order factors plus five select correlated errors; Model 4: correlated content 12 factors plus an orthogonal bifactor; and Model 4A: correlated content 12 factors plus an orthogonal bifactor plus five select correlated errors.

Finally, according to the recommendations of Brookings et al. (2020), two exploratory structural equation models are analyzed, using the method proposed by Asparouhov and Muthén (2009): Model 5 (ESEM): correlated content 12 factors (target oblique rotation) and Model 5A (ESEM): correlated content 12 factors plus a bifactor (i.e., target orthogonal rotation). The comparison of the fit of the models was based on χ^2^, comparative fit index (CFI), Tucker-Lewis Index (TLI), root mean square error of approximation (RMSEA), and standardized root mean square residual (SRMR), using the criteria that indicate that values greater than 0.95 for CFI and TLI would account for an optimal fit and >0.90 would be acceptable; for RMSEA, values under 0.06 would be considered optimal and under 0.8 considered acceptable. For SRMR, the criterion is <0.06 (Hu and Bentler, 1999).

The analyses were carried out using the software Mplus 8.6 (Muthén and Muthén, 1998/2017).

## Results

Table 1 presents the means, standard deviation, asymmetry coefficients, and internal consistency values (i.e., Cronbach’s alpha and omega) for all scales. Cronbach’s alpha was calculated separately for minors and adults as recommended by Gleaves et al. (2014) and evaluated by Brookings et al. (2020). Lower α coefficients were obtained in adolescents in P, IA, and A. In the other subscales, the values would be equivalent.

**TABLE 1:** Descriptive statistics for the EDI-3 scales and composites (*N* = 1,091).

Scale/composite	M	SD	Skew	α total^1^	α low	α high	α 15–18 years old^2^	α 19 and older^3^	ω total	ω low	ω high
Drive for thinness	8.27	7.67	0.89	0.893	0.884	0.902	0.894	0.893	0.905	0.896	0.914
Bulimia	4.98	4.88	1.40	0.746	0.724	0.766	0.730	0.759	0.763	0.742	0.785
Body dissatisfaction	12.37	8.24	0.69	0.816	0.799	0.832	0.813	0.820	0.822	0.807	0.838
Low self-esteem	3.60	3.84	1.19	0.777	0.756	0.796	0.784	0.765	0.788	0.768	0.807
Personal alienation	4.65	4.27	1.39	0.745	0.721	0.767	0.754	0.729	0.740	0.717	0.764
Interpersonal insecurity	7.14	5.40	0.67	0.796	0.777	0.814	0.786	0.805	0.804	0.787	0.822
Interpersonal alienation	6.06	4.23	0.88	0.684	0.655	0.711	0.655	0.710	0.695	0.668	0.723
Interoceptive deficits	7.03	6.05	1.20	0.800	0.781	0.817	0.813	0.782	0.806	0.789	0.823
Emotional dysregulation	5.25	5.10	1.49	0.728	0.703	0.751	0.718	0.737	0.719	0.695	0.744
Perfectionism	8.97	5.12	0.39	0.710	0.682	0.736	0.676	0.739	0.713	0.687	0.739
Asceticism	4.81	3.79	1.09	0.543	0.502	0.582	0.518	0.567	0.552	0.511	0.593
Maturity fears	12.43	6.04	0.69	0.759	0.737	0.780	0.744	0.758	0.766	0.745	0.787

*Scale statistics are based on item sums. For all scales, higher scores reflect greater distress.*

*^1^Total sample (N = 1,091).*

*^2^15–18 years old (N = 503).*

*^3^19 years and older (N = 588).*

The scales that present positive asymmetry are ED (1.49), B (1.40), PA (1.39), ID (1.20), LSE (1.19), and A (1.09), which are expected in most of the subscales when the instrument is applied to a non-clinical sample (Garner, 2004).

Regarding the analysis of the structure of the instrument, the first-order models (1, 1A, and 2) present weak fit to the data, which even worsens when testing the second-order two-factor models. The introduction of a general factor (i.e., bifactor) orthogonal to the 12 factors corresponding to the subscales improves the fit in a relevant way, especially when considering the five pairs of items whose errors would be correlated (Table 2).

**TABLE 2:** Models fit indices.

Model	*X* ^2^	df	CFI	TLI	RMSEA	RMSEA LOW	RMSEA HIGH	SRMR
M0: Null	54329.31**	4,005						
M1: 12 corr factors	12828.50**	3,849	0.822	0.814	0.046	0.045	0.047	0.081
M1A: 12 corr factors, select corr errors (5)	12650.91**	3,844	0.825	0.818	0.046	0.045	0.047	0.081
M2: 12 corr factors, Inconsistency Scale corr errors (10)	12699.25**	3,839	0.824	0.816	0.046	0.045	0.047	0.078
M3: Two 2nd order factors	14251.87**	3,902	0.794	0.789	0.049	0.048	0.050	0.087
M3A: Two 2nd order factors, select corr errors (5)	14096.32**	3,897	0.797	0.792	0.049	0.048	0.050	0.086
M4: Bifactor, corr content factors	9360.91**	3,759	0.893	0.886	0.036	0.035	0.037	0.060
M4A: Bifactor, corr content factors, select corr errors (5)	8915.31**	3,754	0.897	0.890	0.036	0.035	0.036	0.059
M4b: Bifactor, corr content factors, Inconsistency Scale corr errors (10)	8922.83**	3,749	0.897	0.890	0.036	0.035	0.037	0.059
M5 (ESEM): 12 factors. Target oblique rotation	4503.91**	2,991	0.970	0.960	0.022	0.020	0.023	0.028
M5A (ESEM): bifactor: 12 specific factors + general factor. Target orthogonal rotation	4158.66**	2,913	0.975	0.966	0.020	0.018	0.021	0.026
M6: two bifactor corr, 12 corr cont factors	8711.766**	3,758	0.902	0.895	0.035	0.034	0.036	0.059
M7: (ESEM) two bifactor, 12 especific factors, target orthogonal rotation	3932.102**	2,836	0.978	0.969	0.019	0.017	0.020	0.025

**p < 0.01.

*Estimator = Weighted least square mean and variance adjusted estimators (WLSMV).*

*corr, correlated; cont, content.*

However, when evaluating the ESEM models, which do not require that the factorial loads of the items load in a single factor, it is observed how the fit improves substantially, highlighting the 5A model, which considers a bifactor, 12 content factors and target orthogonal rotation.

**FIGURE 1:**
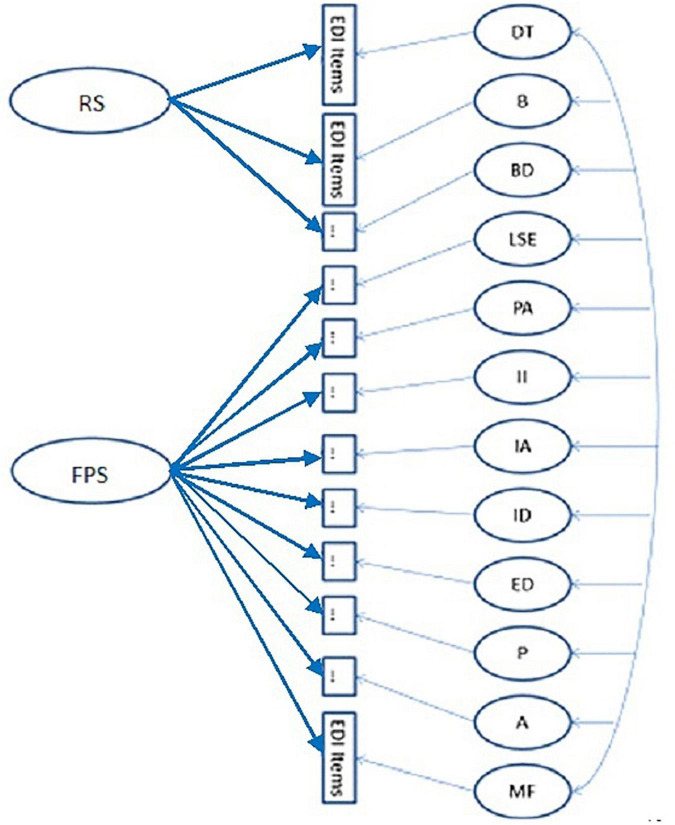
Model 7: two bifactor, 12 specific factors, target orthogonal rotation (ESEM). DT, Drive for thinness; B, Bulimia, BD, Body dissatisfaction; LSE, Low self-esteem; PA; personal alienation; II, Interpersonal insecurity; IA; Interpersonal alienation; ID, Interoceptive deficits; ED, Emotional dysregulation; P, Perfectionism; A, Asceticism; MF, Maturity fears.

Considering the theoretical foundations of EDI-3 (Garner, 2004), which organizes the general structure into two groups of constructs, namely, risk scales and psychological scales, it made more sense to evaluate, rather than a bifactor model with only one general factor, a two-bifactor model, grouping the subscales according to what was proposed by the authors of the original instrument. Model 6 (i.e., two-bifactor model and 12 correlated factors) and model 7, which corresponds to an ESEM two-bifactor model, target orthogonal rotation, were then evaluated, obtaining a noticeable improvement in the adjustment indicators (refer to Table 2 and Figure 1).

When analyzing in detail the outputs of model 7, it is observed (refer to Supplementary Table 1) that almost all the items present significant loads in the general factor (α = 0.05), except for items 72 ED, 81 ED, and 43 P, which, at the same time, present a high association with its specific factor: 0.71^**^, 0.76^**^, and 0.76^**^, respectively.

Regarding the factorial loads of the items by subscales, ten of them do not present a significant load in the expected factor but in their corresponding general factor; these are item 53 of the B subscale (0.54^**^) and item 47 belonging to BD, load in general factor risk scales (0.41^**^); in the PA subscale, items 18, 24, 56, 80, and 84 load significantly in general factor psychological scales (FPS) (0.69^**^, 0.56^**^, 0.77^**^, 0.43^**^, and 0.72^**^, respectively) *; items 54 and 74 of IA load significantly in FPS (0.53^**^ and 0.60^**^); and finally, from subscale A, item 86 presents a higher load of 0.40^**^ in FPS.

There are four items that do not present significant loads > | 0.3| in any subscale and present significant loads in general factor risk scales (RS). These are items 53 of the B subscale (0.54^**^), item 12 BD (0.69^**^), 19 BD (0.61^**^), and 47 BD (0.41^**^).

The same occurs with 20 items that present significant loads in general FPSs and > | 0.3| on no subscale. They are item 41 of LSE (0.64^**^); of the PA subscale, items 18 (0.69^**^), 20 (0.45^**^), 24 (0.56^**^), 56 (0.77^**^), and 84* (0.72^**^). In the II subscale, items 69 (0.57^**^) and 87 (0.49^**^) present this situation; In IA, items 17 (0.49^**^), 30 (0.40^**^), 54 (0.53^**^) 65 (0.51^**^), 74 (0.60^**^), and 76 (0.47^**^). In the ID subscale, items 40 (0.32^**^) and 77 (0.61^**^); in ED, item 67 (0.62^**^) and in A, items 66 (0.63^**^), 78 (0.33^**^), and 86 (0.40^**^).

The reverse situation occurs in a group of three items, that is, they show loads ≤ | 0.3| in the general factor risk scales and significant loads > | 0.3|, in its specific subscale, with values between 0.32 and 0.69. These are items 1 of DT, 5 and 38 of B, and 31 of BD. For the general FPSs, this occurs in ten items with loads between 0.32^**^ and 0.68^**^: items 13, 29, 52, and 63 of P and items 14, 22, 35, 39, 48, and 58 of the subscale MF.

In contrast, items 75 and 88 of A do not present significant loads > | 0.3| in any specific factor, nor in its general factor (GPF). Item 68, also of the A subscale, only loads significantly in the general factor risk scales (0.37^**^).

Finally, item 26 of ID (“I can clearly identify what emotion I am feeling”) presents its highest factor load (0.40^**^) in the II subscale.

## Discussion

In the first place, it should be noted that the analysis carried out revealed a structure congruent with the theoretical postulates of the instrument, in its Spanish version, in a young Chilean non-clinical population.

Regarding the structure of the subscales, it was observed that A and IA have shown the lowest internal consistency, which is consistent with the findings of the Iranian investigation (Dadgostar et al., 2017), with the Spanish and Mexican non-clinical samples (Garner et al., 2010), and with the Swedish and Danish version, in this case, specifically for the A subscale in general population (Clausen et al., 2011; Nyman-Carlsson et al., 2015). None of the items that make up the A subscale presented a load in said factor > | 0.3|.

Also, six items of IA and five of PA presented significant loads only in general FPSs, and six items of MF and 4 of P, only in their specific factor. This is consistent with the analysis of the Spanish version (Garner et al., 2010), wherein IA and PA showed the highest correlations with general FPSs, and MF and P the lowest.

When analyzing the content of the items of the A subscale, it can be observed that some of them explicitly refer to aspects related to a moral dimension (i.e., “moral weakness,” “self-denial,” “suffering to be a better person,” and “human weaknesses”), rather than associated with the initial descriptions of cases of AN and as a specific risk factor for EDs, that are probably not so clearly presented in general population youth (Izydorczyk et al., 2020; Obeid et al., 2021). Something similar happens in IA, where the items are directed toward trusting others, having close friends, and feeling appreciated, which gives the impression of not being perceived in the same dimension by non-clinical youth. This has also been seen in PA, where the content of the items does not appear to be homogeneous, since they include feelings of loneliness and emptiness with elements seemingly related to identity.

In contrast, the P and MF subscales propose apparently much more specific content, which would have a more independent behavior from the rest of the subscales.

This systematic behavior of the items makes it necessary to review these subscales both in relation to the instrument and considering the theoretical postulates.

When comparing the adjustment of the models obtained by Brookings et al. (2020), the adjustment for the Chilean sample is equivalent or slightly lower in those that include 12 correlated factors and among those that consider second-order factors. In contrast, it highlights that when evaluating bifactor models 4 and 4A, the adjustment obtained in the Chilean sample is better than that of the United States sample.

Further, when comparing with the Danish results, the adjustment indices of the Chilean sample are lower for the model of 12 correlated factors for patients and normal controls. This could be attributed to the fact that in this study, a sample of only women was used (Clausen et al., 2011).

As ESEM models are incorporated, it is observed how the adjustment of the models improves, until reaching very good indicators in model 7 (i.e., two–bifactor model, 12 specific factors, and target orthogonal rotation).

Finally, it is interesting to reflect, based on the empirical results, on the cross-loading of the different items and factors and how to consider it, as it would be expected in real-life situations. Therefore, the analyses carried out would allow us to realize that, since latent psychological constructs necessarily interact with each other, the structure of the instruments that evaluate them should adjust to this condition (i.e., ESEM).

### Limitations

A limitation of this study is that the instrument was designed for a clinical population, which limits the variability of responses in a non-clinical sample, with the consequent difficulties for analysis. Therefore, it is necessary to evaluate the fit of the model in a clinical sample.

Furthermore, due to the complexity of the model, it has not been possible to carry out an invariance analysis between men and women, which would have been very interesting to evaluate.

## Data Availability Statement

The raw data supporting the conclusions of this article will be made available by the authors, without undue reservation.

## Ethics Statement

The studies involving human participants were reviewed and approved by the Universidad Adolfo Ibáñez. Written informed consent to participate in this study was provided by the participants’ legal guardian/next of kin.

## Author Contributions

PL-C, CC-M, and FD-C created and organized the study, collected the data, and wrote the first draft of the manuscript. PL-C and JA analyzed and interpreted the data. JA and EC critically reviewed the manuscript and provided the constructive comments. All authors contributed to the article and approved the submitted version.

## Conflict of Interest

The authors declare that the research was conducted in the absence of any commercial or financial relationships that could be construed as a potential conflict of interest.

## Publisher’s Note

All claims expressed in this article are solely those of the authors and do not necessarily represent those of their affiliated organizations, or those of the publisher, the editors and the reviewers. Any product that may be evaluated in this article, or claim that may be made by its manufacturer, is not guaranteed or endorsed by the publisher.
