# Supplementary material for: Psychometric Properties of the Eating Disorder Inventory-3 (EDI-3) in Chilean Youth
Source: Front Psychol. 2022 Mar 1;13:806563. doi: 10.3389/fpsyg.2022.806563 (PMC8923287; doi:10.3389/fpsyg.2022.806563)
Supplement: Supplementary file 1 [file Table_1.docx]

*Supplementary Table 1. Factor loadings for two-bifactor ESEM orthogonal rotation*

| **Item*** | **General factor Risk Scales** | **General factor Psych Scales** | **DT** | **B** | **BD** | **LSE** | **PA** | **II** | **IA** | **ID** | **ED** | **P** | **A** | **MF** | **Uniqueness** |  |
| --- | --- | --- | --- | --- | --- | --- | --- | --- | --- | --- | --- | --- | --- | --- | --- | --- |
| 1 (r) | **.21**** | .05 | **.43**** | -.29** | .09** | -.03 | .17** | -.01 | .05 | -.12** | -.02 | -.06 | .15* | -.02 | .60** |  |
| 7 | **.79**** | .16** | **.31**** | .02 | .01 | -.09** | .00 | -.02 | .00 | .06* | -.01 | -.06** | .02 | .04 | .24** |  |
| 11 | **.69**** | .24** | **.40**** | .09** | -.02 | -.02 | -.04 | -.05* | -.06 | .06* | -.02 | .01 | .08** | -.01 | .28** |  |
| 16 | **.67**** | .14** | **.48**** | .06** | -.03 | -.02 | -.09* | -.01 | -.01 | .15** | .02 | .14** | -.06 | .03 | .25** |  |
| 25 | **.67**** | .21** | **.50**** | .04 | .01 | .07* | -.05 | -.05* | .00 | .04 | .03 | .08** | -.04 | -.01 | .24** |  |
| 32 | **.84**** | .18** | **.30**** | -.02 | .03 | .03 | .05* | -.05* | -.02 | .12** | -.04* | .04* | .00 | .01 | .15** |  |
| 49 | **.68**** | .18** | **.53**** | .06** | .03 | .01 | -.03 | -.06** | -.02 | .11** | .04 | .08** | -.01 | .04 | .19** |  |
| 4 | **.31**** | .24** | .10** | **.55**** | -.01 | .03 | .05 | -.12** | .01 | .13** | -.07* | .04 | -.08* | .02 | .49** |  |
| 5 | **.21**** | .14** | -.23** | **.54**** | -.13** | -.05 | .03 | -.03 | -.05 | .07* | -.02 | .03 | -.13** | .01 | .55** |  |
| 28 | **.31**** | .15** | -.12** | **.55**** | -.08* | -.06 | -.07* | .00 | -.03 | .05 | .04 | .08* | .00 | -.02 | .54** |  |
| 38 | **.25**** | .17** | -.13** | **.69**** | -.07* | -.08** | -.05 | .04 | .01 | .04 | .09** | .06 | .03 | .02 | .39** |  |
| 46 | **.33**** | .27** | .04 | **.49**** | -.01 | .04 | -.13** | .07* | -.04 | .04 | .22** | .05 | .10 | .07* | .49** |  |
| 53 | **.54**** | .36** | .17** | **.08** | .05 | .10 | .00 | .00 | .02 | -.09 | .18** | .01 | .25** | -.01 | .43** |  |
| 61 | **.37**** | .32** | .06 | **.35**** | -.03 | .07 | .11 | .00 | .00 | .06 | .12** | .02 | .20** | -.06 | .56** |  |
| 64 | **.50**** | .28** | .32** | **.51**** | .04 | -.01 | .15** | -.06 | .05 | .14** | -.02 | .07* | .13* | -.04 | .24** |  |
| 2 | **.69**** | .18** | -.31** | .11** | **-.27**** | .09** | -.01 | -.02 | -.03 | -.03 | .05 | .07* | -.11** | .03 | .28** |  |
| 9 | **.69**** | .13** | .05 | -.11** | **.38**** | -.06* | -.09* | -.01 | -.09* | .15** | -.06* | .00 | .03 | .06* | .30** |  |
| 12 (r) | **.69**** | .23** | -.15** | -.05 | **-.13**** | .14** | .13** | .09** | .09* | -.11** | .08** | -.08** | -.04 | -.01 | .35** |  |
| 19 (r) | **.61**** | .38** | .03 | -.06* | **.13**** | .25** | .21** | .06* | .08 | -.13** | -.07** | -.09** | -.02 | .01 | .32** |  |
| 31 (r) | **.14**** | .33** | -.07* | .07* | **.32**** | .24** | .17** | .08* | .08 | -.18** | -.04 | -.06 | .01 | .05 | .63** |  |
| 45 | **.67**** | .15** | .08** | -.06* | **.44**** | -.02 | -.18** | -.01 | -.03 | .04 | .05 | .01 | .00 | .01 | .29** |  |
| 47 | **.41**** | .21** | .22** | .16** | **-.01** | -.07 | -.09 | .02 | -.08 | .10** | .07 | .03 | -.07 | .07* | .67** |  |
| 55 (r) | **.48**** | .23** | -.09** | -.07** | **.61**** | .11** | .18** | .10** | .02 | -.02 | -.09** | -.03 | -.03 | .05* | .27** |  |
| 59 | **.55**** | .07* | .02 | -.07 | **.34**** | -.07 | -.15** | -.07 | .00 | .05 | .09* | -.03 | .08 | -.02 | .52** |  |
| 62 (r) | **.49**** | .23** | -.08** | -.02 | **.54**** | .13** | .07 | .05 | .16** | -.09** | -.03 | -.05 | -.12** | .06* | .33** |  |
| 10 | .15** | **.61**** | -.04 | .02 | .01 | **.30**** | -.13** | -.05 | -.12* | .03 | -.04 | -.02 | .00 | .04 | .47** |  |
| 27 | .20** | **.62**** | -.01 | -.01 | .01 | **.43**** | -.12** | -.10** | -.12* | .09** | -.09** | .00 | .05 | .01 | .34** |  |
| 37 (r) | .23** | **.59**** | .06* | -.03 | .05 | **.41**** | .10* | .17** | .02 | -.03 | -.12** | -.12** | -.11** | .03 | .35** |  |
| 41 | .40** | **.64**** | .04 | -.04 | .06 | **.25**** | .07 | -.14** | .00 | .09** | -.12** | .07* | .03 | -.01 | .31** |  |
| 42 (r) | .04 | **.46**** | .03 | .03 | .11** | **.53**** | .10* | .14** | .04 | -.11** | .00 | -.27** | -.01 | .04 | .38** |  |
| 50 (r) | .02 | **.53**** | -.09** | -.10** | .10** | **.46**** | .17* | .14** | .21** | -.12** | .01 | -.15** | -.06 | .05* | .34** |  |
| 18 | .09** | **.69**** | -.06 | -.03 | -.08* | -.05 | **.01** | -.08* | .23** | .10* | -.14** | -.04 | .12* | -.04 | .39** |  |
| 20 (r) | .16** | **.45**** | .05 | .01 | .06 | .24** | **.19*** | .16** | .12* | -.07* | -.01 | -.15** | -.18** | .07* | .57** |  |
| 24 | .24** | **.56**** | .05 | .01 | .09* | .29** | **-.03** | -.07 | .11* | .03 | -.04 | .05 | .02 | .10** | .50** |  |
| 56 | .05 | **.77**** | -.09* | -.02 | -.04 | -.02 | **.03** | -.07 | .12 | .16** | -.04 | -.03 | .19** | .02 | .31** |  |
| 80 (r) | -.02 | **.43**** | -.04 | .08* | .13** | .09* | **.12** | .13** | .43** | -.10** | -.01 | -.13** | -.23** | -.03 | .48** |  |
| 84 | .22** | **.72**** | .06* | .04 | -.02 | .21** | **-.05** | -.12** | -.14** | .05 | .10* | .07* | -.07 | .10** | .32** |  |
| 91 (r) | .09** | **.59**** | -.03 | .03 | .08* | .22** | **.30**** | .16** | .15* | .06 | -.09** | -.05 | -.06 | .00 | .43** |  |
| 15 (r) | -.01 | **.42**** | -.06* | -.01 | -.02 | .10** | .18** | **.73**** | -.03 | .09** | -.06* | .01 | -.03 | -.03 | .23** |  |
| 23 (r) | -.05 | **.51**** | .04 | .01 | .08** | .05 | -.06 | **.52**** | .13** | -.09** | -.07* | -.11** | -.10* | -.01 | .41** |  |
| 34 | .04 | **.45**** | -.08* | -.03 | -.14** | -.05 | -.05 | **.48**** | -.09* | .32** | -.03 | .05 | .04 | .05 | .41** |  |
| 57 (r) | -.02 | **.34**** | -.04 | -.04 | .09** | .12** | .16** | **.56**** | .11* | -.04 | .06 | -.01 | -.02 | .00 | .50** |  |
| 69 (r) | -.04 | **.57**** | -.05 | .02 | .15** | .05 | .02 | **.29**** | .23** | -.19** | -.06 | -.18** | -.09* | -.02 | .43** |  |
| 73 (r) | -.11** | **.49**** | .01 | .00 | .07* | .01 | -.08 | **.52**** | .19** | -.16** | -.14** | -.13** | -.11* | -.09** | .35** |  |
| 87 | -.01 | **.49**** | -.10** | -.04 | -.06 | -.25** | -.17 | **.16**** | -.01 | -.10* | -.08* | .03 | .25** | -.05 | .55** |  |
| 17 (r) | -.03 | **.49**** | .08* | .01 | .02 | -.10** | .09* | .29** | **.18**** | -.14** | -.04 | -.03 | -.01 | .03 | .59** |  |
| 30 (r) | -.08* | **.40**** | .00 | .02 | .11** | .09* | .12 | .24** | **.28**** | -.25** | -.04 | -.08* | .10* | -.03 | .59** |  |
| 54 | .04 | **.53**** | .01 | .04 | .04 | -.20** | -.07 | .16** | **-.04** | -.08 | -.05 | .04 | .28** | -.09* | .54** |  |
| 65 | .22** | **.51**** | .03 | .01 | -.05 | -.18** | -.02 | -.10** | **.16**** | .17** | .03 | .03 | .09 | -.01 | .58** |  |
| 74 | .00 | **.60**** | .02 | .03 | -.01 | -.06 | .02 | .03 | **.02** | .03 | .05 | .11** | .26** | -.01 | .56** |  |
| 76 (r) | .06 | **.47**** | -.12** | -.03 | .08** | -.10** | .10 | .23** | **.30**** | -.05 | -.09** | -.03 | -.12** | .01 | .57** |  |
| 89 (r) | -.03 | **.60**** | -.02 | -.04 | .08* | .20** | .11 | .15** | **.43**** | -.14** | -.02 | -.07* | -.12 | -.05 | .34** |  |
| 8 | .22** | **.48**** | .17** | .06 | -.04 | -.09* | .01 | -.15** | .04 | **.40**** | -.01 | .00 | .03 | .04 | .50** |  |
| 21 | .10** | **.48**** | .06* | .03 | -.03 | .00 | -.04 | .05 | -.10** | **.55**** | .01 | -.05 | -.04 | -.02 | .44** |  |
| 26 (r) | -.01 | **.35**** | -.05 | .06 | .07* | .14** | .20** | .40** | .02 | **.13**** | .10** | -.09** | -.14* | .06* | .59** |  |
| 33 | .20** | **.54**** | -.04 | .05 | -.01 | .07* | -.01 | .02 | .03 | **.40**** | -.03 | -.03 | .06 | .05 | .49** |  |
| 40 | .33** | **.32**** | .03 | .28** | -.01 | .03 | .00 | .05 | -.03 | **.19**** | .07 | .10** | .00 | -.02 | .66** |  |
| 44 | .10** | **.55**** | .17** | .07* | .02 | -.11** | .01 | -.09** | .02 | **.48**** | .10** | .09** | -.04 | -.01 | .38** |  |
| 51 | .10** | **.49**** | .11** | .13** | .05 | .03 | -.06 | -.01 | -.16** | **.39**** | .15** | .05 | -.01 | .02 | .51** |  |
| 60 | .09** | **.54**** | -.03 | .08** | -.02 | -.03 | -.01 | .06* | -.09** | **.58**** | .02 | .02 | .05 | -.04 | .34** |  |
| 77 | .06 | **.61**** | -.05 | .06 | -.01 | -.07 | .06 | -.10** | -.03 | **.22**** | .15** | .03 | .11** | .03 | .52** |  |
| 67 | .15** | **.62**** | .06 | .05 | .01 | -.11** | -.02 | -.11** | -.01 | .11** | **.15*** | .00 | -.14** | -.06 | .51** |  |
| 70 | .13** | **.36**** | .03 | .08* | .02 | -.05 | .01 | -.08* | -.19** | .10* | **.30**** | .12** | -.18** | .08** | .65** |  |
| 72 | .03 | **.07** | -.12** | .06 | -.03 | .13** | -.05 | .01 | .27** | .13** | **.71**** | .02 | .26* | .08* | .29** |  |
| 79 | .04 | **.52**** | .02 | -.04 | -.02 | -.17** | .08 | -.21** | -.22** | -.05 | **.45**** | .07* | -.16* | -.12** | .35** |  |
| 81 | .09** | **.05** | -.05 | .10** | -.08* | .07 | -.05 | .11** | .16* | .15** | **.76**** | .01 | .23* | .08* | .28** |  |
| 83 | .09** | **.50**** | .07 | .05 | .01 | -.22** | -.03 | -.14** | -.32** | -.09 | **.40**** | .11** | -.32** | -.05 | .28** |  |
| 85 | .17** | **.65**** | .11** | .08* | .01 | -.22** | .05 | -.17** | -.21** | -.02 | **.32**** | -.02 | -.18* | -.01 | .27** |  |
| 90 | .00 | **.62**** | .12* | .01 | -.03 | .00 | -.01 | -.04 | .00 | -.04 | **.38**** | -.04 | .33** | -.01 | .34** |  |
| 13 | -.01 | **.20**** | -.02 | -.01 | .03 | -.02 | -.01 | -.01 | .06 | -.02 | .07* | **.61**** | -.01 | .01 | .57** |  |
| 29 | .10** | **.12**** | .04 | -.01 | .00 | -.05 | -.05 | -.11** | .04 | .07* | -.06* | **.57**** | .06 | .08** | .61** |  |
| 36 | .12** | **.34**** | .14** | .18** | -.07* | .02 | .02 | .03 | -.16** | -.01 | .09* | **.47**** | .06 | .03 | .56** |  |
| 43 | .06 | **.04** | -.01 | -.03 | -.05 | -.09** | -.01 | -.10** | .15** | .07* | .04 | **.76**** | -.06 | .05 | .37** |  |
| 52 | .10** | **.20**** | .11** | .14** | -.01 | -.15** | -.09 | -.03 | -.16** | .00 | .02 | **.48**** | .08 | .01 | .62** |  |
| 63 | -.01 | **.08*** | .00 | .16** | -.07* | -.18** | .05 | -.03 | -.18** | -.08* | .03 | **.43**** | .20** | -.12** | .65** |  |
| 66 | .20** | **.63**** | .06 | .02 | -.04 | .12** | -.03 | -.08** | .00 | .12** | .07* | .19** | **.09*** | -.08* | .47** |  |
| 68 | .37** | **.25**** | .14** | .11** | .03 | -.07* | -.07 | -.07* | -.06 | .10** | .05 | .18** | **.14**** | .04 | .69** |  |
| 75 | .11** | **.11**** | -.06 | .11** | -.05 | -.10* | -.02 | -.16** | -.02 | .05 | .07 | .13** | **.28**** | -.02 | .81** |  |
| 78 | .26** | **.33**** | .19** | .13** | .03 | .16** | .02 | -.03 | .03 | .03 | .20** | .08* | **.14*** | -.04 | .68** |  |
| 82 | -.03 | **.30**** | .07 | .06 | .02 | .04 | -.05 | -.03 | -.15** | -.08 | .15** | .21** | **.19**** | -.02 | .76** |  |
| 86 | .23** | **.40**** | .13** | .15** | .08 | -.01 | -.05 | .06 | -.17** | -.08 | .17** | .08 | **.06** | .13** | .64** |  |
| 88 | .00 | **.29**** | -.01 | .07 | -.01 | -.16** | -.15 | -.12** | -.01 | .01 | .15** | .01 | **.30**** | -.07 | .74** |  |
| 3 | .21** | **.48**** | .00 | .12** | .04 | .18** | -.19** | -.04 | .06 | .10** | -.01 | .09** | .00 | **.52**** | .35** |  |
| 6 | .06 | **.39**** | .01 | .10* | .00 | .11* | -.22** | -.01 | .03 | -.01 | .01 | -.01 | .10 | **.41**** | .60** |  |
| 14 | -.04 | **.13**** | -.01 | .00 | .07* | -.03 | -.22** | -.12** | -.03 | .03 | .08** | .15** | .00 | **.58**** | .55** |  |
| 22 (r) | -.01 | **.08*** | .06 | -.06 | .02 | -.04 | .32** | .01 | .01 | -.04 | .00 | -.11** | .00 | **.68**** | .42** |  |
| 35 | .09** | **.23**** | .04 | .06 | -.04 | -.06 | -.15** | .03 | -.06 | .17** | .03 | .21** | .02 | **.32**** | .73** |  |
| 39 (r) | .01 | **.16**** | .02 | -.07* | .03 | .04 | .18** | .02 | -.04 | -.08** | .00 | -.12** | -.09 | **.53**** | .62** |  |
| 48 | .09** | **.30**** | -.06 | .04 | .00 | -.07* | -.27** | -.11** | -.03 | .05 | .03 | .17** | .02 | **.58**** | .43** |  |
| 58 (r) | .03 | **.21**** | .02 | -.07* | .03 | .02 | .32** | .12** | .02 | -.09** | -.03 | -.16** | -.06 | **.66**** | .36** |  |
| *Note*. * *p* < .05; ** *p* < .01. DT = Drive for Thinness; B = Bulimia; BD = Body Dissatisfaction; LSE = Low Self-Esteem; PA = Personal Alienation; II = Interpersonal Insecurity; IA = Interpersonal Alienation; ID = Interoceptive Deficits; ED = Emotional Dysregulation; P = Perfectionism; A = Asceticism; MF = Maturity Fears. Bolded values indicate the factor where the item theoretically belongs. | | | | | | | | | | | | | | |  |  |
|  |  |  |  |  |  |  |  |  |  |  |  |  |  |  |  |  |
|  |  |  |  |  |  |  |  |  |  |  |  |  |  |  |  |  |
